# Effects of water flow treatment on muscle quality, nutrient composition and volatile compounds in common carp (*Cyprinus carpio*)

**DOI:** 10.1016/j.fochx.2025.102257

**Published:** 2025-02-04

**Authors:** Lei Wang, Lingran Wang, Chang Liu, Di Feng, Jintai Huang, Zhan Jin, Fangran Ma, Jiaxin Xu, Yuyue Xu, Meng Zhang, Miao Yu, Hongxia Jiang, Zhigang Qiao

**Affiliations:** a College of Fisheries, Henan Normal University, Xinxiang, 453007, China; b Engineering Technology Research Center of Henan Province for Aquatic Animal Cultivation, Henan Normal University, Xinxiang, 453007, China; c Engineering Lab of Henan Province for Aquatic Animal Disease Control，Henan Normal University, Xinxiang, 453007, China; d Observation and Research Station on Water Ecosystem in Danjiangkou Reservoir of Henan Province, Nanyang 474450, China

**Keywords:** *Cyprinus carpio*, Water flow, GC-IMS, Volatile substances, Muscle quality, Nutrient composition

## Abstract

This study explored the effects of water flow on the muscle quality, nutrient composition, and volatile compounds in *Cyprinus carpio*. Fish were exposed to three treatments: sustained water flow (SG, 1 bl/s, 24 h/d), intermittent water flow (IG, 1 bl/s, 8 h/d), and control group (CG, 3 cm/s). Results indicated that SG improved water-holding capacity, muscle fiber density, hardness, and chewiness, while IG enhanced gumminess, springiness, and resilience. Nutritionally, CG exhibited higher crude lipid content and the highest levels of ΣSFA. Conversely, SG showed elevated ∑EPA + DHA and ω-3 fatty acid levels compared to IG. Volatile compound analysis demonstrated that CG contained higher levels of aldehydes and alcohols, associated with off-flavors, whereas IG and SG produced fresher and sweeter aroma profiles, enhancing sensory quality. These findings provide valuable insights into improving the muscle quality, nutritional value, flavor characteristics, processing, and preservation of common carp through water flow treatment.

## Highlights

• Sustained water flow significantly increases beneficial fatty acids (EPA and DHA) in common carp.
• Intermittent water flow improves muscle elasticity, while sustained flow enhances muscle hardness.
• Water flow intensity influences muscle quality, including water-holding capacity and fiber density.
• Water flow positively impacts volatile flavor compounds, reducing undesirable odors and enhancing aroma in fish muscle.

## 1 Introduction

Aquatic foods are among the most nutritious and widely consumed sources of high-quality protein globally, accounting for approximately 15 % of total animal protein intake. In 2022, global fisheries and aquaculture production reached 223.2 million tonnes, underscoring the increasing importance of aquaculture in ensuring food security (FAO, 2024). However, the rapid expansion of the aquaculture industry has introduced significant challenges, particularly the transition from solely on production to meeting market demands for superior-quality fish products. Traditional pond-based aquaculture methods, while prevalent, often contribute to environmental degradation, including water pollution and eutrophication. These issues negatively impact fish quality, leading to poor texture, undesirable off-flavors, and reduced nutritional value (Munni et al., 2015). Consequently, the aquaculture sector urgently requires the adoption of green and sustainable development strategies to ensure long-term growth and improved product quality.

One promising approach is the Recirculating Aquaculture System (RAS), a innovative and intensive aquaculture model designed to maximize water resource utilization and recycling through advanced recirculation and filtration systems. RAS mitigates environmental impacts, enhances farming efficiency, and significantly improves the quality of aquaculture products (Espinal & Matulić, 2019). Studies have demonstrated that RAS enhances the muscle quality and nutritional composition of various aquatic species, including common carp (*Cyprinus carpio*) (Ma et al., 2023), largemouth bass (*Micropterus salmoides*) (Jia et al., 2022) and yellow catfish (*Pelteobagrus fulvidraco*) (Zhang et al., 2022). Previous research by our team revealed that water flow in RAS induces swimming behavior in common carp, which enhances muscle properties, nutrient composition, and flavor profiles (Ma et al., 2023; Wang et al., 2022). This finding underscores the importance of regulating water flow velocity in RAS to stimulate fish swimming exercise, providing a foundation for further investigation into the effects of water flow on muscle growth and quality.

Recent studies have identified swimming exercise induced by water flow as a critical factor influencing muscle growth and development, with direct effects on fish texture and nutritional value (Timmerhaus et al., 2021). Adequate water flow induces significant physiological adaptations in fish muscles, promoting effective swimming performance and enhancing muscle quality. Research indicates that swimming at optimal velocities can lead to higher growth rates and improved muscle development (Rodgers & Gomez Isaza, 2023). This flow-induced exercise triggers adaptive changes in muscle fibers, including hypertrophy (enlargement of existing muscle fibers) and hyperplasia (an increase in the number of muscle fibers), resulting in alterations in muscle fiber diameter and density. These changes significantly influence muscle texture, a key determinant of consumer acceptance and product quality (Ibarz et al., 2011). Furthermore, water flow treatment impacts the chemical composition of muscle by increasing concentrations of essential and umami-flavored amino acids, reducing fat content, and optimizing the fatty acid profiles. These biochemical changes substantially enhance fish muscle quality (Li et al., 2016; Song et al., 2012). Flavor, a critical determinant of consumer acceptance, plays a vital role in aquatic food quality. The distinctive flavor profiles of aquatic animals, shaped by factors such as aquaculture practices (Ma et al., 2023), feed composition (Bruni et al., 2020), and water pollution (Han et al., 2024), remarkably influence consumer preferences. Understanding and managing these factors are crucial for enhancing flavor quality in aquaculture products. Notably, exercise training has been shown to shape the flavor profiles of fish muscle (Harimana et al., 2019).

Common carp, one of the most widely cultured freshwater fish species, accounted for approximately 4.2 million tonnes of global aquaculture production in 2020 (FAO, 2022). As a species with strong swimming ability and adaptability to diverse environmental conditions, common carp are particularly suitable for studies investigating the effects of water flow on muscle properties (Martin & Johnston, 2006). While existing research has predominantly examined the effects of varying water flow velocities, limited attention has been given to the influence of different durations and intensities of water flow exposure on fish muscle quality. This study seeks to address this gap by investigating the effects of various water flow treatments on muscle quality, nutrient composition, and volatile flavor compounds in common carp within a RAS. The findings provide valuable insights into the relationship between water flow treatment and muscle quality, offering practical implications for improving the nutritional value, sensory attributes, processing, and preservation of common carp. Ultimately, these insights contribute to the production of higher-quality aquatic food products.

## 2 Material and methods

### 2.1 Experimental fish and design

**Fig. 1:**
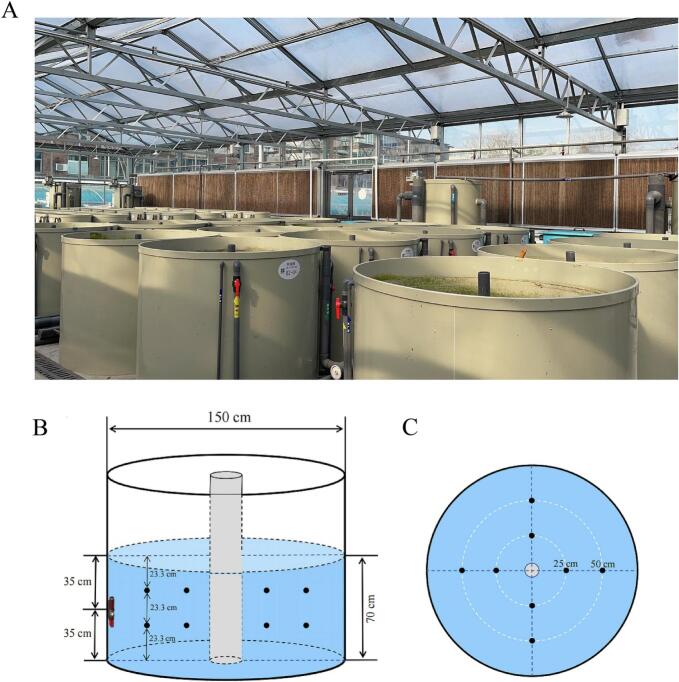
Recirculating Aquaculture System and schematic of measurement points in the cultivation tank. A: Recirculating Aquaculture System; B: Front view of the cultivation tank; C: Top view of the cultivation tank.

In this study, 270 healthy common carp were obtained from Xingda Aquaculture Farm, Zhengzhou City, Henan Province, China. The fish had an average body length of 13.61 ± 0.19 cm and an average body weight of 63.91 ± 1.45 g. The fish were randomly divided into three groups: the Sustained water flow treatment [SG, 1 bl/s (body length per second), 24 h/d], the Intermittent water flow treatment (IG, 1 bl/s, 8 h/d, from 9:00 AM to 5:00 PM), and a Control group (CG, with an actual flow velocity of 3 cm/s in the tank), each consisting of three replicates with 30 fish per replicate, and the experimental lasted 90 days. The experimental setup employed a RAS at the Aquaculture Base of the College of Fisheries, Henan Normal University (Fig. 1A). Throughout the experiment, the water temperature was maintained between 24 and 26 °C, pH levels ranged from 7.16 to 8.67, and dissolved oxygen levels were kept at 7–9 mg/L. Fish were fed puffed compound feed (Tongwei Co., Ltd., Xinxiang, China) containing 35 % crude protein, 7 % crude fiber, 3 % crude fat, 1.1 % total phosphorus, 1.8 % lysine, 16 % ash, and 12 % moisture. Feeding was conducted twice daily at 9:00 AM and 5:00 PM. In the experimental groups (SG and IG), flow velocity was regulated using a variable-frequency wave-making pump, while in the CG group, flow velocity was maintained at 3 cm/s through aeration devices and water circulation within the tank. Flow speed was measured at 16 uniform points in the fish's active area using an LS300-A micro flow meter and the average value was calculated to represent the flow velocity of the culture tank (Fig. 1B-C). Additionally, fish length and weight were measured monthly, with flow velocity adjusted based on the average length of the fish. At the beginning of the experiment, the average body length of all fish was 13.6 cm, and the initial flow velocity was set to 13.6 cm/s (approximately 1 bl/s). On day 28, the second adjustment was made, with the average body lengths of the CG, IG, and SG groups showing no significant differences (*P* > 0.05). As a result, the water flow velocity for all groups was adjusted to 16 cm/s (approximately 1 bl/s). On day 56, a third adjustment was performed: the SG group had an average body length of 22 cm, and its water flow velocity was set to 22 cm/s. The IG and CG groups had average body lengths of 21.44 cm and 21.20 cm, respectively, and their water flow velocities were set to 21 cm/s, corresponding to approximately 1 bl/s (Table S1).

### 2.2 Sample collection

All procedures in this study were conducted in strict compliance with the Guidance on Treating Experimental Animals (2006) issued by the Ministry of Science and Technology of the People's Republic of China, as well as the Regulations for the Administration of Affairs Concerning Experimental Animals (Order No. 2 of the State Science and Technology Commission, 1988). Furthermore, the study adhered to the guidelines established by the Science Research Experiment Ethics Committee at Henan Normal University (ethical approval code: HNSD-SCXY-2116BS1066), ensuring that animal suffering and stress were minimized throughout the experimental process. After 90 days of cultivation, all experimental fish (*N* = 270) were fasted for 24 h. A total of 21 common carp per group (seven per replicate) were selected and anesthetized using 100 mg/L tricaine methane sulfonate (MS-222). Muscle samples from the dorsal region were collected on ice for subsequent analysis. Specifically, three fish per group were used to assess muscle water-holding capacity (WHC), muscle tissue sections, and muscle proximate composition. Six fish per group were used for muscle texture characteristics analysis. Additionally, nine fish per group, with every three samples pooled into one biological replicate, were used to analyze muscle amino acid and fatty acid contents. Finally, three fish per group, with 20 g of dorsal muscle per fish, were pooled for GC-IMS analysis.

### 2.3 Analysis of the muscle tissue sections

For the analysis of muscle tissue sections, approximately 100 mg of dorsal muscle tissue was collected from each group. The samples were immersed in 4 % fixative solution (P1110, Solarbio Corp., Beijing, China) to prepare for section analysis. Following fixation, the tissues were dehydrated, embedded in paraffin wax, sectioned, and stained using Hematoxylin and Eosin (HE) staining techniques. Muscle fiber structure, diameter, and density were subsequently analyzed using Caseviewer software.

### 2.4 WHC

WHC was assessed following the methodologies and equations described by Jia et al. (2022), with appropriate modifications for the experimental materials. Six indices of muscle WHC were measured: liquid loss, storage loss, freeze-thaw leakage, drip loss, cooking loss, and centrifugal loss. Each parameter was evaluated using 10 g muscle samples.

### 2.5 Texture characteristics

Texture characteristics were evaluated using modified methods adapted from Wei et al. (2016) and Jia et al. (2022). For the analysis, dorsal muscle samples were cut into 2.0 cm cubes. The texture characteristics of both raw and cooked muscles were measured using a TA-XT Plus texture analyzer. The parameters measured included hardness, gumminess, springiness, chewiness, shear force, cohesiveness, and resilience. Cooked muscle samples were prepared by boiling for 5 min, followed by cooling prior to analysis.

### 2.6 Determination of muscle chemical composition

#### 2.6.1 Proximate composition of the muscle

The proximate composition of the muscle was analyzed as follows: moisture content was determined using a constant-temperature drying oven set at 105 °C (GB 5009.3–2016). Ash content was measured by incinerating the samples in a muffle furnace at 550 °C until a constant weight was achieved (GB 5009.4–2016). Crude lipid content was assessed using the Soxhlet extraction method (GB 5009.6–2016), while crude protein content was quantified employing the Kjeldahl method (GB 5009.5–2016).

#### 2.6.2 Analysis of muscle amino acid and fatty acid contents

Fatty acid content was determined using a 7890B Gas Chromatograph (Agilent Technologies, USA), with calculations performed using the area normalization method (GB 5009.168–2016). Amino acid content was analyzed using an Amino Acid Analyzer A300 (GB 5009.124–2016), also employing the area normalization method for primary amino acids. Tryptophan content was not assessed due to its degradation during acid hydrolysis.

### 2.7 GC-IMS analysis

Three samples were taken from each barrel, with one fish selected from each of the three parallel groups to form a composite sample. This process yielded three biological replicates for each group. For each replicate, 20 g of dorsal muscle was collected from each fish to create the mixed samples. Volatile flavor compounds were analyzed and identified using a GC-IMS instrument (FlavorSpec®, G.A.S., Germany), following the methodology described by Ma et al. (2023).

### 2.8 Data analysis

Statistical analysis was performed using SPSS 26.0 software. One-way ANOVA followed by Tukey's honestly significant difference (HSD) test, was applied to identify significant differences at a 95 % confidence level. A significance threshold of *P* < 0.05 was used to denote statistically significant differences, while *P* > 0.05 indicated non-significant differences. Results were reported as mean ± standard deviation (SD). GraphPad Prism 9.3.0 and Excel 2021 were employed for graphing and data analysis.

## 3 Results and discussion

### 3.1 Muscle tissue sections

**Fig. 2:**
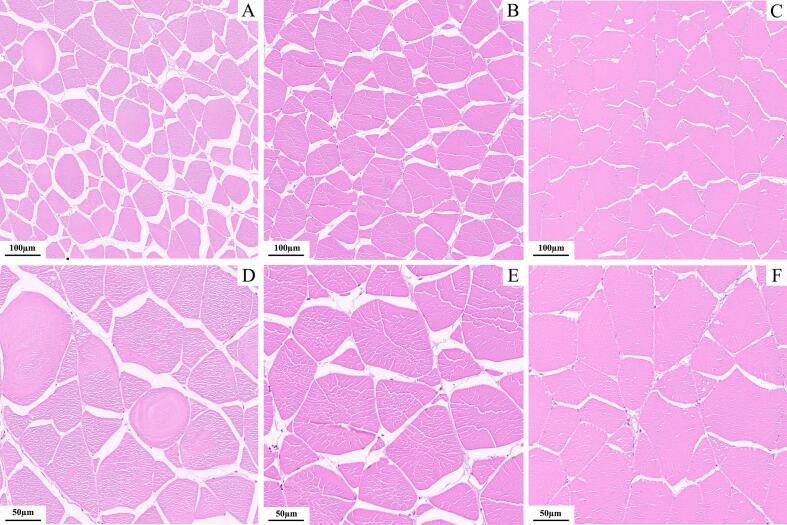
Cross-sectional microscopic images of common carp muscle fibers in treatment groups with different water flow. A, D: 100 μm, 50 μm for CG group; B, E: 100 μm, 50 μm for IG group; C, F: 100 μm, 50 μm for SG group.

Unlike mammals, fish experience both hyperplasia and hypertrophy during growth, with water flow-induced exercise serving as a crucial stimulus for these processes. As shown in Table S2 and Fig. 2, no significant differences were observed in muscle fiber short diameter and long diameter among the treatment groups. However, the SG group exhibited significantly higher muscle fiber density (214.44 ± 5.09 n/mm^2^) compared to both the CG (165.55 ± 13.47 n/mm^2^) and IG (174.44 ± 10.18 n/mm^2^) groups (*P* < 0.05), suggesting that sustained water flow exercise promoted muscle fiber density primarily through hyperplasia rather than hypertrophy. Previous studies have demonstrated that sustained water flow can increase growth hormone (GH) levels in fish, which plays a regulatory role in muscle protein synthesis, contributing to both muscle fiber hyperplasia and hypertrophy (Barrett & Mckeown, 1988; Shrivastava et al., 2018; Vélez et al., 2017). While hypertrophy is typically the dominant mechanism in muscle growth following short-term exercise (Ibarz et al., 2011), hyperplasia, can persist for extended periods in rapidly growing fish species (Rowlerson & Veggetti, 2001). Based on a review of previous studies, the three-month duration of this experiment suggests that prolonged water flow treatment likely increased GH levels, thereby promoting muscle fiber hyperplasia and leading to the observed increase in muscle fiber density in the SG group. Additionally, high-intensity exercise may induce muscle fiber damage and apoptosis in fish. These stress conditions could activate the proliferation and differentiation of muscle satellite cells, further promoting muscle fiber hyperplasia (Chang & Rudnicki, 2014). Nonetheless, these findings remain preliminary and speculative. Further research is needed to investigate the specific mechanisms through which water flow-induced exercise influences muscle development in fish, particularly the relationship between GH level changes, muscle fiber hyperplasia, and hypertrophy.

### 3.2 WHC

Freshwater fish muscle typically contains 75–80 % water, much of which is retained in intra- and extra-myofibrillar spaces. Changes in intracellular architecture can significantly affect water retention (Diao et al., 2021; Huff-Lonergan & Lonergan, 2005). WHC, which refers to the muscle's capacity to retain moisture during processing, is a critical quality indicator for meat (Szmańko et al., 2021). WHC is evaluated through liquid loss, storage loss, freeze-thaw leakage, drip loss, and centrifugal loss, which are negatively correlated with WHC, while cooking loss is positively correlated (Warner, 2014). In this study, no significant differences were observed among the treatment groups regarding centrifugal loss, drip loss, storage loss, liquid loss, or freeze-thaw leakage (*P* > 0.05). However, the SG group had the highest cooking loss at 80.83 ± 0.81 % which was significantly higher than that of the IG group (76.80 ± 0.96 %) (*P* < 0.05) (Table S3). These results suggest that the muscle from the SG group retained more moisture during cooking, reflecting a higher WHC. The increased WHC in the SG group is likely attributable to the higher muscle fiber density induced by sustained water flow treatment, which created a denser mesh structure among muscle cells, enabling them to trap and retain more water (Li et al., 2012). Consequently, the enhanced WHC likely contributed to a juicier and more tender texture, benefiting subsequent processing and storage.

### 3.3 Texture characteristics

Texture is a critical determinant of the palatability and eating quality of fish muscle, influenced by parameters such as hardness, gumminess, cohesiveness, chewiness, shearing, and resilience (Cheng et al., 2014). Among these, hardness is particularly significant, as it serves as a key indicator of texture acceptability in aquatic products (Liu et al., 2021). The texture of muscle is shaped by various factors including muscle fiber diameter, fiber arrangement, collagen content, and the distribution of intramuscular connective tissue (Nishimura, 2010). Sustained water flow treatment, which stimulates exercise, has been identified as an effective method for improving these texture characteristics (Cai et al., 2023).

**Fig. 3:**
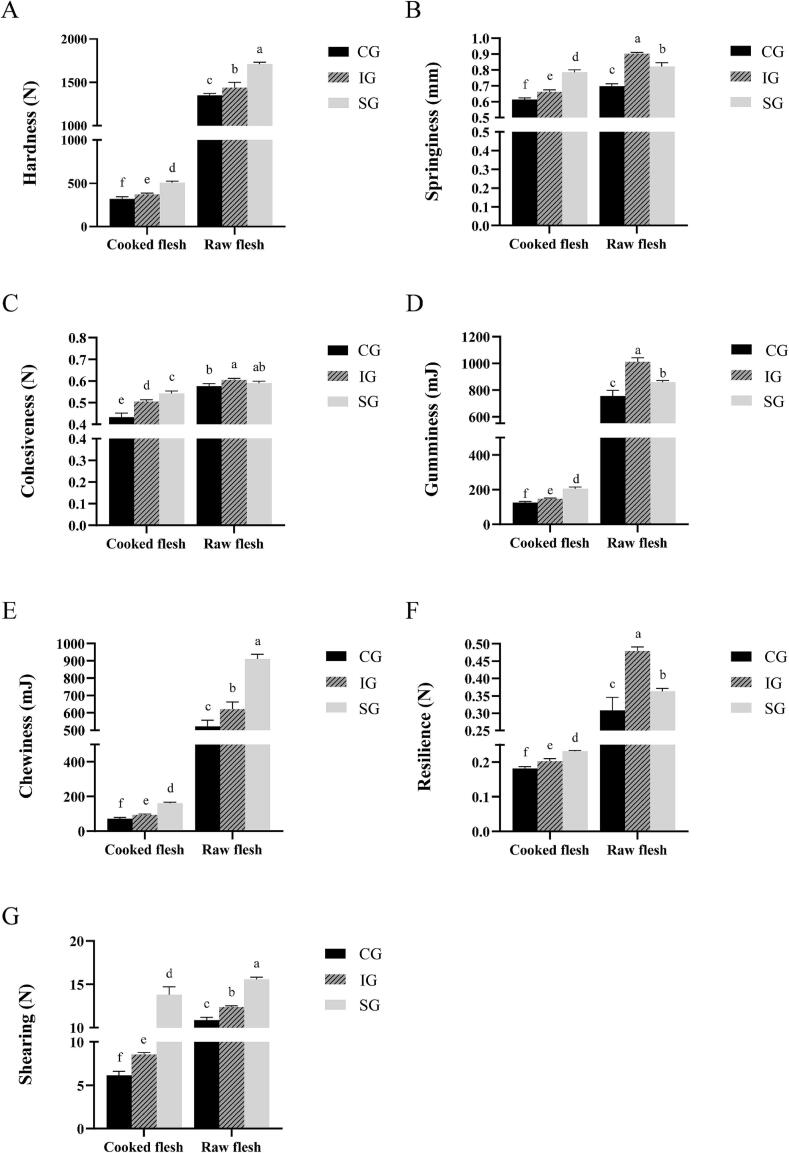
Texture characteristics of common carp muscle in treatment groups with different water flow. A: hardness; B: springiness; C: cohesiveness; D: gumminess; E: chewiness; F: resilience; G: shearing. Values represent means ± SD (*n* = 6). The same letter denotes no significant difference, and different letters indicating significant differences (*P* < 0.05).

In this study, the SG group exhibited significantly greater hardness, chewiness, and shearing in raw flesh compared to the other groups (*P* < 0.05). In contrast, the IG group demonstrated the highest cohesiveness, surpassing the CG group significantly (*P* < 0.05). Additionally, the IG group showed superior springiness, gumminess, and resilience, significantly exceeding the other two groups (*P* < 0.05) (Fig. 3). These findings are consistent with the results of Li et al. (2016), who found a positive correlation between muscle texture and fiber density. Higher muscle fiber density, accompanied by reduced muscle fiber diameter, typically contributes to enhanced muscle hardness (Johnston et al., 2000). In our study, both the SG and IG groups showed higher muscle fiber density (Fig. 2), which likely explains the observed texture differences. Specifically, the SG group displayed firmer texture traits, such as increased hardness, chewiness, and shearing. On the other hand, despite similar muscle fiber density, the IG group had more extensive intermyofibrillar spaces. The springiness and resilience of muscle tissue are often linked to the structure of these spaces (Lin et al., 2009), which likely contributed to the enhanced elasticity, springiness, gumminess, and resilience observed in the IG group. These structural differences suggest that the SG group's muscle is firmer, while the IG group's muscle exhibited superior elasticity, both of which are crucial for the overall texture and eating quality of fish muscle.

The texture of cooked fish flesh is influenced by several factors, including the size of muscle fiber after heating, interstitial spaces, the volume of coagulated proteins, and the formation of collagen and lipid gels (Ayala et al., 2005; Ayala et al., 2010). As shown in Fig. 3, the hardness, springiness, cohesiveness, chewiness, gumminess, resilience, and shearing of common carp muscle were significantly reduced after cooking compared to raw flesh (*P* < 0.05). However, for cooked flesh, the SG group exhibited significantly higher values for all texture parameters compared to both the CG and IG groups (*P* < 0.05). This suggests that the SG group's muscle retained superior texture characteristics after cooking. These improvements are likely due to the denaturation and contraction of muscle fibers, sarcoplasmic proteins, and the coagulation of proteins during the cooking process. In conclusion, the SG group's muscle exhibited superior texture in both raw and cooked forms, likely due to the increased muscle fiber density. The IG group demonstrated enhanced elasticity and resilience, likely attributed to the larger intermyofibrillar spaces observed. Further research on the microstructure of both raw and cooked fish muscle tissues is necessary to fully understand the mechanisms behind these texture improvements.

### 3.4 Muscle chemical composition

#### 3.4.1 Proximate composition of the muscle

Between 96 % and 98 % of fish muscle consists of water, crude protein, crude lipid, and ash—collectively referred to as the “proximate composition” of fish. These components, along with the amino acid profile, fatty acid composition, and mineral content, are crucial for the overall nutritional evaluation of fish (Ahmed et al., 2022). Water flow-induced exercise in fish is an energy-intensive process that increases the utilization of carbohydrates and fats to meet energy demands (Li et al., 2015). As shown in Table S4, the SG group had the lowest muscle moisture content at 75.57 ± 0.99 %, which was significantly lower than the CG (77.47 ± 0.12 %) and IG (76.84 ± 0.19 %) groups (*P* < 0.05). Conversely, the SG group exhibited the highest crude protein content at 20.19 ± 0.40 %, which was significantly greater than that of the CG group (18.60 ± 0.41 %) (*P* < 0.05). For crude lipid content, the CG group had the highest value (14.45 ± 0.77 %), which was significantly greater than both the IG (11.83 ± 0.39 %) and SG (11.93 ± 1.37 %) groups (*P* < 0.05). These findings suggest that water flow treatment enhances energy expenditure, reduces fat accumulation in muscles, and promotes protein accumulation. Similar results were observed in Chinese perch (*Siniperca chuatsi*) following sustained swimming at different water flow velocities (Zhu et al., 2023). We hypothesize that the reduction in crude lipid content in the SG group may be attributed to increased lipid oxidation during exercise, which promotes fat breakdown. Additionally, the SG group likely experienced greater glycolysis and lipid oxidation during swimming, further reducing crude fat content (Magnoni et al., 2013). Like other animals, fish require essential amino acids from their diet for protein synthesis (Wilson, 2003). Under the conditions of this experiment, another study published by our team (Wang et al., 2025) showed that, despite all groups being fed the same diet, there were no significant differences in feed conversion ratio (FCR) among the three groups. However, the SG group exhibited significantly higher muscle crude protein content compared to the CG group, suggesting that sustained swimming exercise may enhance the SG group's ability to absorb essential amino acids from feed, thereby promoting protein synthesis. Nonetheless, this study did not assess the metabolic responses of the fish post-exercise, indicating the need for further research to confirm this hypothesis.

#### 3.4.2 Fatty acid composition analysis

**Fig. 4:**
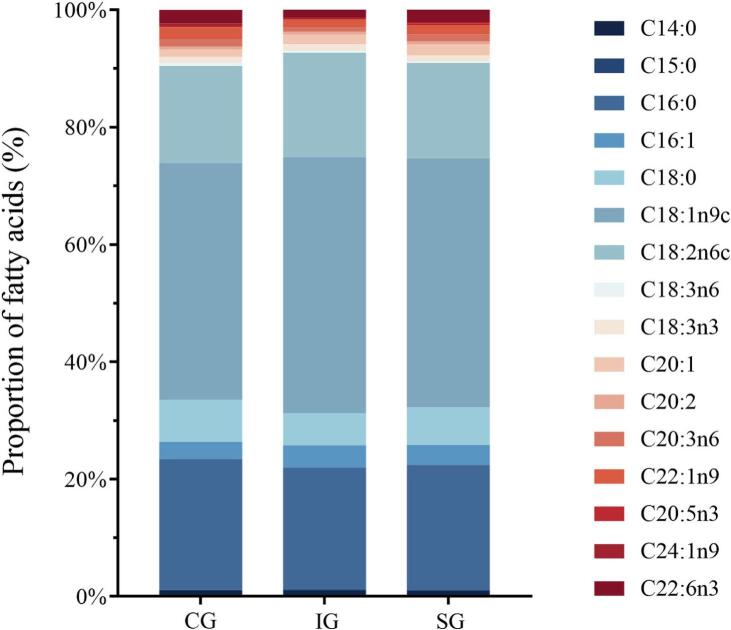
Fatty acid content of common carp in treatment groups with different water flow. The bar graph represents the percentage of fatty acid content.

The composition and concentration of fatty acids in fish muscle play a crucial role in determining its nutritional value and palatability. Due to the complexity of energy metabolism, water flow-induced exercise has been shown to regulate the fatty acid content in fish muscle (Rasmussen et al., 2011). To assess the effects of different water flow intensities on the fatty acid composition of common carp muscle, we analyzed the fatty acid content across the treatment groups. As shown in Fig. 4 and Table S5, we identified a total of 16 fatty acids including 4 saturated fatty acids (ΣSFA), 5 monounsaturated fatty acids (ΣMUFA), and 7 polyunsaturated fatty acids (ΣPUFA). Among these, ΣPUFA, including ω-3, ω-6, EPA, and DHA, are particularly important for human health, making their content a key factor in evaluating the nutritional value of fish (Kapoor et al., 2021). Previous studies have suggested that low-intensity exercise can increase ΣPUFA content in fish muscle, while high-intensity exercise may reduce these levels (Kiessling et al., 2005; Li et al., 2016). However, in our study, no significant differences in ΣPUFA content were observed the treatment groups, indicating that exercise under moderate water flow velocities and stress intensities does not reduce ΣPUFA content in fish muscle. Notably, the ΣEPA + DHA and ω-3 fatty acid content in the SG group were significantly higher than those in the IG group (*P* < 0.05). Specifically, ΣEPA + DHA levels were 2.31 ± 0.16 % in the SG group, significantly greater than in the IG group (1.46 ± 0.02 %). Similarly, ω-3 fatty acid content was 3.33 ± 0.24 % in the SG group, significantly higher than in the IG group (2.60 ± 0.02 %). These results suggest that sustained water flow treatment is more effective in preserving EPA + DHA and ω-3 levels in muscle, which are beneficial for human health. In contrast, the CG group exhibited the highest ΣSFA content (30.62 ± 1.62 %), which was significantly higher than that of the IG group (27.45 ± 0.52 %) (*P* < 0.05). Additionally, the CG group had the lowest ΣMUFA content (46.58 ± 1.08 %), which was significantly lower than that of the IG (50.58 ± 0.36 %) and SG (50.00 ± 2.11 %) groups (*P* < 0.05). ΣSFA are key components of the fatty acid profile in fish, and numerous studies recommend replacing them with ΣPUFA to reduce the risk of cardiovascular disease (Heileson, 2020; Joris & Mensink, 2024). Although previous studies have not shown a significant effect of water flow treatment on ΣSFA concentration in fish muscle (Harimana et al., 2019), our results suggest that exercise training significantly reduces ΣSFA content, thereby improving the nutritional quality of muscle. These findings emphasize the complexity of fatty acid metabolism in fish muscle, with differences between this study and previous research potentially attributable to variations in species, experimental conditions, and the diverse effects of exercise intensity on fatty acid metabolism.

#### 3.4.3 Amino acid composition analysis

The amino acid composition of fish plays a vital role in determining the nutritional quality of fish protein, especially with regard to essential amino acids (EAAs) and those contributing to flavor. In this study, we analyzed the muscle of common carp from different treatment groups, identifying 17 common amino acids (excluding tryptophan), which included 9 essential amino acids (EAA), 4 delicious or flavorful amino acids (DAA), and 4 non-essential amino acids (NEAA), as shown in Table 1. Previous studies have suggested that moderate water flow can enhance the amino acid composition of fish muscle (Li et al., 2016; Song et al., 2012). However, in our study, no significant differences were observed in the total content of ∑EAA, ∑NEAA, and ∑DAA among the treatment groups (*P* > 0.05). Nonetheless, certain individual amino acids showed significant differences. Specifically, the IG group exhibited significantly higher serine content (3.76 ± 0.14 %) than the CG group (3.43 ± 0.10 %) (*P* < 0.05). Furthermore, the arginine content was significantly higher in the CG (5.98 ± 0.01 %) and IG groups (5.98 ± 0.07 %) compared to the SG group (5.84 ± 0.07 %) (*P* < 0.05). These findings suggest that intermittent water flow treatment may help preserve specific amino acids, such as serine and arginine, by reducing their use for energy metabolism in comparison to sustained water flow treatment. While the overall amino acid profile in muscle tissue remained largely unaffected by the different water flow treatments, these findings underscore the necessity of further research to explore the underlying mechanisms. A deeper understanding of how water flow intensity influences amino acid metabolism could provide valuable insights into optimizing water flow conditions to improve the nutritional quality of fish, particularly in terms of their amino acid composition.

**Table 1:** Amino acid content of common carp in treatment groups with different water flow (% of total amino acid content, dry matter, *n* = 3).

Amino acids	CG	IG	SG
Asp^☆^	10.51 ± 0.06	10.45 ± 0.08	10.59 ± 0.09
Glu^☆^	15.35 ± 0.05	15.19 ± 0.10	15.32 ± 0.10
Ser	3.43 ± 0.10^b^	3.76 ± 0.14^a^	3.60 ± 0.08^ab^
Arg^★^	5.98 ± 0.01^a^	5.98 ± 0.07^a^	5.84 ± 0.07^b^
Gly^☆^	5.36 ± 0.19	5.33 ± 0.25	5.11 ± 0.21
Thr^★^	4.14 ± 0.08	4.24 ± 0.09	4.17 ± 0.13
Pro	3.76 ± 0.09	3.74 ± 0.14	3.61 ± 0.10
Ala^☆^	6.15 ± 0.06	6.09 ± 0.11	6.11 ± 0.03
Val^★^	5.46 ± 0.05	5.36 ± 0.09	5.43 ± 0.05
Met^★^	3.45 ± 0.04	3.48 ± 0.10	3.46 ± 0.03
Cys	1.11 ± 0.04	1.16 ± 0.04	1.19 ± 0.10
Ile^★^	4.93 ± 0.13	4.95 ± 0.07	4.99 ± 0.03
Leu^★^	8.38 ± 0.07	8.36 ± 0.05	8.42 ± 0.06
Phe^★^	4.98 ± 0.06	4.84 ± 0.13	5.02 ± 0.06
His^★^	3.49 ± 0.06	3.50 ± 0.03	3.55 ± 0.06
Lys^★^	10.15 ± 0.06	10.11 ± 0.09	10.17 ± 0.07
Tyr	3.38 ± 0.05	3.45 ± 0.03	3.44 ± 0.03
∑EAA	50.95 ± 0.21	50.84 ± 0.40	51.05 ± 0.23
∑NEAA	49.05 ± 0.21	49.16 ± 0.40	48.95 ± 0.23
∑DAA	37.36 ± 0.14	37.06 ± 0.19	37.12 ± 0.25

★ indicates Essential Amino Acids (EAAs). ☆denotes Delicious or Flavorful Amino Acids. ∑EAA represents the Total Amount of Essential Amino Acids. ∑NEAA signifies the Total Amount of Non-Essential Amino Acids. ∑DAA refers to the Total Amount of Delicious or Flavorful Amino Acids. The same letter denotes no significant difference, and different letters indicating significant differences (*P* < 0.05).

### 3.5 GC-IMS analysis

#### 3.5.1 Principal component analysis (PCA)

**Fig. 5:**
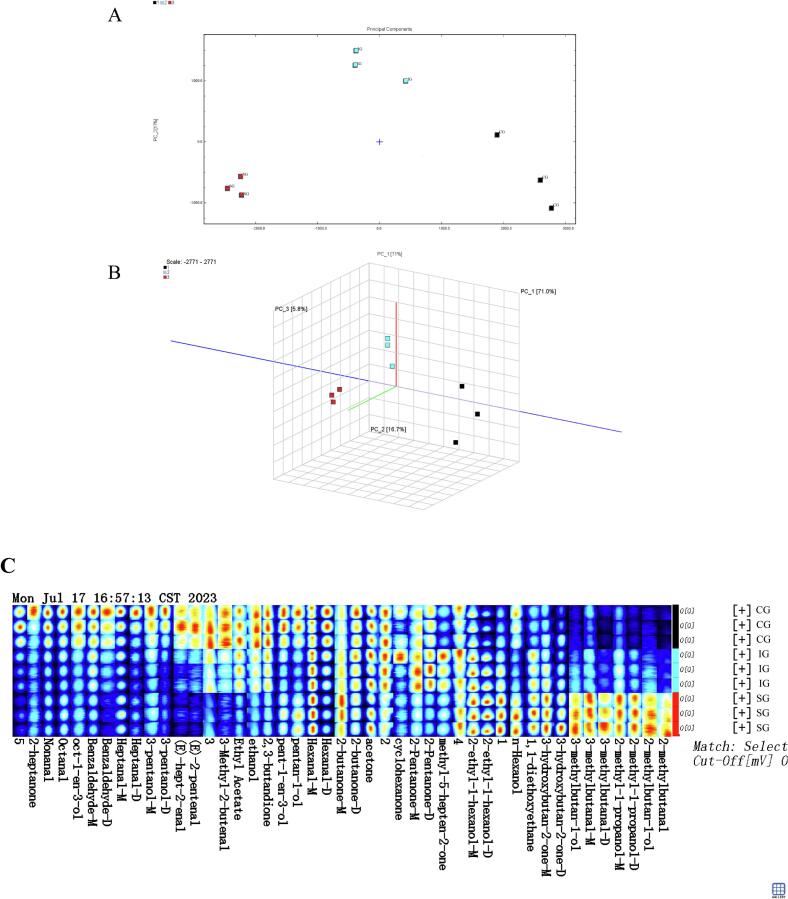
Volatile substance analysis of common carp in treatment groups with different water flow. A: two-dimensional PCA score plot.; B: three-dimensional PCA score plot; C: Fingerprint spectra of selected volatile compounds from the GC-IMS (Each row in the figure represents all selected signal peaks from one sample. Each column corresponds to the signal peaks of the same volatile compound across different samples. Compounds followed by -M or —D indicate the monomer and dimer forms of the same substance, respectively. Peaks with numeric identifiers are unidentified.).

Principal Component Analysis (PCA) was conducted to evaluate the variations in volatile compounds present in the muscle of common carp across the three treatment groups. The results, illustrated in Fig. 5A and B, revealed significant differences and clear separations among the groups, indicating distinct differentiation in the principal components associated with volatile compounds across the treatment groups.

#### 3.5.2 Comparative analysis of volatile compounds spectrum

Fig. S1 presents the GC-IMS spectrum of volatile compounds in the muscle of common carp from different treatment groups. The blue background and the red vertical line at the 1.0 mark on the abscissa represent the normalized Reaction Ion Peak (RIP). Each point near the RIP peak corresponds to a specific volatile organic compound, with the color gradient indicating peak intensity. The ordinate displays the retention time (in seconds) from gas chromatography, while the abscissa represents the relative migration time (normalized, arbitrary units). The spectrum reveals distinct differences in the volatile compounds' profiles across the three treatment groups.

To facilitate a more intuitive comparison of volatile substances in the muscle of common carp among the treatment groups, the CG sample spectrum was used as the baseline reference. Spectra from the IG and SG groups were subtracted from this reference to generate a differential comparison chart (Fig. S2). A white background indicates identical concentrations of volatile organic compounds in both samples, while red hues represent higher concentrations in the target sample, and blue hues indicate lower concentrations. These findings highlight significant changes in the content of flavor compounds in the fish muscle, which are associated with increased water flow intensity.

#### 3.5.3 Identification and differential analysis of volatile components in muscle

Fingerprint spectra provide a means to compare differences in flavor compound content among the three groups based on peak signal intensity, creating a unique fingerprint pattern for each sample. Fig. 5C offers an intuitive visualization of the differences and trends in volatile components across the groups. Due to variations in concentration and changes in proton affinity, certain flavor compounds may produce multiple signals, manifesting as both monomers and dimers. The GC-IMS technique was employed to measure volatile components in fish muscle, with qualitative spectra presented in Fig. S3. A total of 45 volatile components were detected across the three sample types, of which 40 were identified (Table S6). These comprised 1 ester, 11 ketones, 14 aldehydes, and 14 alcohols among the volatile compounds. Notably, concentrations of Nonanal, Octanal, Oct-1-en-3-ol, Benzaldehyde, Heptanal, Hexanal, Pentan-1-ol, 3-pentanol, Pent-1-en-3-ol, (*E*)-2-pentenal, and (E)-hept-2-enal were significantly higher in the CG group compared to the other two groups (*P* < 0.05). Similarly, the SG group exhibited significantly elevated levels of 2-ethyl-1-hexanol-M, Hexanol, 3-hydroxybutan-2-one-M, 3-methylbutanal, 2-methyl-1-propanol, and 2-methylbutan-1-ol compared to the other two groups (*P* < 0.05). Volatile compounds are critical to the flavor and overall organoleptic qualities of fish (Qiu et al., 2023). Alcohols and aldehydes, the primary volatile compounds in fish, are largely produced through the oxidation of PUFAs (Li et al., 2023). Among these, aldehydes significantly influence flavor due to their lower odor thresholds, while alcohols, with higher odor thresholds, have a less pronounced effect (Cheng et al., 2023). Freshwater fish typically exhibit a plant-like aroma that is subtler and more pleasant compared to the stronger, more pronounced odors of saltwater fish. However, the elevated aldehyde content in freshwater fish can lead to more prominent fishy and earthy flavors (An et al., 2020; Wu et al., 2022; Zhou et al., 2024).

In this study, the CG group displayed significantly higher aldehyde and alcohol content compared to the IG and SG groups. The aldehydes and alcohols such as nonanal, octanal, heptanal, pent-1-en-3-ol, and (E)-hept-2-enal in the CG group are associated with greasy and fishy odors originating from lipid oxidation (Alasalvar et al., 2005). Furthermore, compounds including hexanal, heptanal, oct-1-en-3-ol, and pentan-1-ol are linked to earthy flavor and green aroma (Liu et al., 2024). The IG group exhibited elevated concentrations of ketones, particularly methyl-5-hepten-2-one and 2-pentanone. Methyl-5-hepten-2-one imparts a citrus odor (Hallier et al., 2005), while 2-pentanone contributes to a savory fish flavor (Mao et al., 2023). Conversely, the SG group contained diverse alcohols, aldehydes, and ketones, including sweet-flavored compounds such as 3-hydroxybutanone, 3-methylbutanal, and 2-methylbutan-1-ol, which are by products of free sugars decomposition in tissues (Jones et al., 2022).

The observed differences in volatile compound profiles in this study can be attributed to fatty acid oxidation, a key process in flavor generation in fish muscle (Huang et al., 2022). Water flow treatment has been shown to reduce the levels of unsaturated fatty acids in fish muscle, which are more prone to oxidation than ΣSFA. It also alters the ratio of unsaturated to saturated fatty acids, promoting the oxidation of the more readily oxidizable compounds and thereby decreasing the generation of undesirable flavor compounds (Ganesan et al., 2014; Li et al., 2016; Schönfeld & Wojtczak, 2008). During exercise, the heightened rate of fatty acid oxidation fish muscle to meet energy demands results in more complete oxidation of fatty acids, thereby minimizing the accumulation of intermediate metabolites, such as peroxides and aldehydes, which are associated with undesirable flavors (Feng et al., 2021; Muscella et al., 2020). Exercise further enhances oxygen consumption and facilitates the exchange of lipophilic substances between the fish and its environment, expelling volatile compounds with undesirable flavors and reducing fishy odors (Schram et al., 2016). In summary, water flow-induced exercise modifies the volatile compounds in the muscles of common carp, thereby improving the flavor quality of the fish.

## 4 Conclusion

This experiment examined the influence of water flow treatment on the muscle quality, nutrient composition, and volatile compounds in common carp. The findings demonstrated that sustained water flow treatment significantly enhanced muscle WHC, increased muscle fiber density, and elevated the levels of EPA + DHA and ω-3 fatty acids. Additionally, the treatment enhanced muscle texture by increasing springiness and hardness, while reducing fat deposition, decreasing ΣSFA content, and increasing ΣMUFA levels. These modifications collectively improved the nutritional value and edible quality of common carp. Moreover, water flow treatment enhanced sensory attributes by reducing undesirable odors and intensifying aroma. These findings underscore water flow treatment as a promising aquaculture strategy to meet the rising consumer demand for nutritious and flavorful fish products.

## Ethical statement

All procedures in this study were conducted in strict accordance with the Guidance on Treating Experimental Animals (2006) issued by the Ministry of Science and Technology of the People's Republic of China, the Regulations for the Administration of Affairs Concerning Experimental Animals (Order No. 2 of the State Science and Technology Commission, 1988). Additionally, the study adhered to the guidelines of the Science Research Experiment Ethics Committee at Henan Normal University (ethical approval code: HNSD-SCXY-2116BS1066), ensuring that animal suffering and stress were minimized during the experiments.

## CRediT authorship contribution statement

**Lei Wang:** Writing – original draft, Validation, Supervision, Resources, Project administration, Investigation, Funding acquisition. **Lingran Wang:** Writing – review & editing, Writing – original draft, Methodology, Formal analysis, Data curation, Conceptualization. **Chang Liu:** Software. **Di Feng:** Visualization. **Jintai Huang:** Investigation. **Zhan Jin:** Investigation. **Fangran Ma:** Investigation. **Jiaxin Xu:** Data curation. **Yuyue Xu:** Data curation. **Meng Zhang:** Methodology. **Miao Yu:** Methodology. **Hongxia Jiang:** Formal analysis. **Zhigang Qiao:** Conceptualization.

## Declaration of competing interest

The authors declare that they have no known competing financial interests or personal relationships that could have appeared to influence the work reported in this paper.

## Data Availability

The data that support the findings of this study are available from the corresponding author upon reasonable request.

## Appendix A Supplementary data

Supplementary material: Three figures of volatile compounds and Supplementary tables. Image 1
