# Supplementary material for: Effects of water flow treatment on muscle quality, nutrient composition and volatile compounds in common carp (Cyprinus carpio)
Source: Food Chem X. 2025 Feb 4;26:102257. doi: 10.1016/j.fochx.2025.102257 (PMC11848482; doi:10.1016/j.fochx.2025.102257)
Supplement: Supplementary file 1 — Supplementary material: Three figures of volatile compounds and Supplementary tables. [file mmc1.docx]

**Supplementary Figures：**

**Table S1**

Monthly length and weight measurements during the aquaculture period.

| Measurement | Group | 0 days | 28 days | 56 days |
| --- | --- | --- | --- | --- |
| Body length | CG | 13.59 ± 1.10 | 15.80 ± 1.36 | 21.20 ± 1.74^b^ |
|  | IG | 13.61 ± 1.14 | 16.03 ± 1.36 | 21.44 ± 2.06^b^ |
|  | SG | 13.44 ± 1.16 | 16.14 ± 1.28 | 22.04 ± 1.97^a^ |
| Body weight | CG | 63.40 ± 13.60 | 113.08 ± 28.97^b^ | 253.07 ± 69.62^b^ |
|  | IG | 65.54 ± 15.03 | 123.07 ± 29.99^ab^ | 263.56 ± 75.78^ab^ |
|  | SG | 62.78 ± 15.09 | 123.99 ± 32.34^a^ | 292.01 ± 85.12^a^ |

Values represent means ± SD.

The same letter denotes no significant difference, and different letters indicating significant differences (*P* < 0.05).

**Table S2**

| Items | CG | IG | SG |
| --- | --- | --- | --- |
| Short diameter (µm) | 89.63 ± 8.91 | 83.17 ± 10.80 | 69.27 ± 15.37 |
| Long diameter (µm) | 132.43 ± 9.321 | 130.43 ± 11.44 | 119.33 ± 11.42 |
| Density (n/mm^2^) | 165.55 ± 13.47^b^ | 174.44 ± 10.18^b^ | 214.44 ± 5.09^a^ |

Long diameter, short diameter, and density of common carp muscle in treatment groups with different water flow intensities (n = 3).

Values represent means ± SD.

The same letter denotes no significant difference, and different letters indicating significant differences (*P* < 0.05).

**Table S3**

Analysis of WHC in common carp muscle among treatment groups with different water flow intensities (%, n = 3).

| Items | CG | IG | SG |
| --- | --- | --- | --- |
| Centrifugal loss | 5.84 ± 0.25 | 5.22 ± 0.58 | 5.63 ± 0.77 |
| Drip loss | 5.74 ± 1.23 | 4.09 ± 0.52 | 4.52 ± 0.74 |
| Liquid loss | 21.77 ± 1.24 | 23.18 ± 0.92 | 21.62 ± 0.36 |
| Storage loss | 3.28 ± 0.41 | 2.90 ± 0.50 | 3.40 ± 0.36 |
| Freeze thaw leakage | 3.28 ± 0.09 | 2.95 ± 0.09 | 3.17 ± 0.29 |
| Cooking loss | 79.58 ± 0.14^ab^ | 76.80 ± 0.96^b^ | 80.83 ± 0.81^a^ |

Values represent means ± SD.

The same letter denotes no significant difference, and different letters indicating significant differences (*P* < 0.05)

**T****able S4**

General nutrients of common carp in treatment groups with different water flow intensities (%, n = 3).

| Items | CG | IG | SG |
| --- | --- | --- | --- |
| Moisture | 77.47 ± 0.12^a^ | 76.84 ± 0.19^a^ | 75.57 ± 0.99^b^ |
| Ash | 5.24 ± 0.09 | 4.98 ± 0.24 | 4.90 ± 0.27 |
| Crude lipid | 14.45 ± 0.77^a^ | 11.83 ± 0.39^b^ | 11.93 ± 1.37^b^ |
| Crude protein | 18.60 ± 0.41^b^ | 19.63 ± 0.72^ab^ | 20.19 ± 0.40^a^ |

Values represent means ± SD.

The same letter denotes no significant difference, and different letters indicating significant differences (*P* < 0.05)

**Table S5**

Fatty acid content of common carp in treatment groups with different water flow intensities (%, n = 3, dry matter).

| Fatty acids | CG | IG | SG |
| --- | --- | --- | --- |
| C14:0 | 0.99 ± 0.03^a^ | 1.01 ± 0.03^a^ | 0.92 ± 0.00^b^ |
| C15:0 | 0.15 ± 0.02 | 0.15 ± 0.00 | 0.15 ± 0.01 |
| C16:0 | 22.26 ± 0.89 | 20.71 ± 0.39 | 21.28 ± 0.48 |
| C16:1 | 2.94 ± 0.19^b^ | 3.75 ± 0.12^a^ | 3.41 ± 0.42^ab^ |
| C18:0 | 7.19 ± 0.77^a^ | 5.43 ± 0.08^b^ | 6.37 ± 0.35^ab^ |
| C18:1n9c | 40.28 ± 1.80 | 43.46 ± 0.30 | 42.31 ± 2.39 |
| C18:2n6c | 16.55 ± 0.96 | 17.68 ± 0.19 | 16.17 ± 1.45 |
| C18:3n6 | 0.57 ± 0.17^a^ | 0.34 ± 0.01^b^ | 0.30 ± 0.01^b^ |
| C18:3n3 | 0.98 ± 0.07 | 1.13 ± 0.01 | 1.01 ± 0.09 |
| C20:1 | 1.29 ± 0.07^c^ | 1.67 ± 0.10^b^ | 1.88 ± 0.09^a^ |
| C20:2 | 0.43 ± 0.03 | 0.42 ± 0.03 | 0.49 ± 0.03 |
| C20:3n6 | 1.31 ± 0.29 | 0.83 ± 0.04 | 1.14 ± 0.19 |
| C22:1n9 | 2.02 ± 0.52 | 1.19 ± 0.10 | 1.62 ± 0.31 |
| C20:5n3 (EPA) | 0.29 ± 0.05 | 0.22 ± 0.00 | 0.27 ± 0.04 |
| C24:1n9 | 0.33 ± 0.09 | 0.23 ± 0.01 | 0.27 ± 0.03 |
| C22:6n3 (DHA) | 2.32 ± 0.38^a^ | 1.24 ± 0.02^b^ | 2.04 ± 0.14^a^ |
| ∑SFA | 30.62 ± 1.62^a^ | 27.45 ± 0.52^b^ | 28.83 ± 0.84^ab^ |
| ∑MUFA | 46.58 ± 1.08^b^ | 50.58 ± 0.36^a^ | 50.00 ± 2.11^a^ |
| ∑PUFA | 22.47 ± 0.32 | 21.98 ± 0.23 | 21.50 ± 1.86 |
| EPA+DHA | 2.62 ± 0.43^a^ | 1.46 ± 0.02^b^ | 2.31 ± 0.16^a^ |
| ω-3 | 3.60 ± 0.37^a^ | 2.60 ± 0.02^b^ | 3.33 ± 0.24^a^ |
| ω-6 | 18.45 ± 0.57 | 18.94 ± 0.23 | 17.67 ± 1.61 |

The same letter denotes no significant difference, and different letters indicating significant differences (*P* < 0.05)

**Table S6**

Qualitative results of GC-IMS in common carp muscle from treatment groups with different exercise intensities.

| Count | Compound | CAS# | Formula | MW | RI | Rt [sec] | Dt [RIPrel.] | Peak intensity | | | Comment |
| --- | --- | --- | --- | --- | --- | --- | --- | --- | --- | --- | --- |
|  |  |  |  |  |  |  |  | CG | IG | SG |  |
| 1 | Nonanal | C124196 | C_9_H_18_O | 142.2 | 1099.1 | 769.544 | 1.48531 | 575.14 ± 58.58^a^ | 368.80 ± 42.38^b^ | 364.28 ± 16.96^b^ |  |
| 2 | 2-ethyl-1-hexanol-M | C104767 | C_8_H_18_O | 130.2 | 1038.1 | 645.391 | 1.41881 | 2244.62 ± 17.62^c^ | 3094.21 ± 77.51^b^ | 3381.54 ± 92.83^a^ | Monomer |
| 3 | 2-ethyl-1-hexanol-D | C104767 | C_8_H_18_O | 130.2 | 1037.7 | 644.751 | 1.80452 | 574.70 ± 10.07^c^ | 1133.56 ± 87.92^b^ | 1442.29 ± 63.38^a^ | Dimer |
| 4 | Octanal | C124130 | C_8_H_16_O | 128.2 | 998.8 | 576.275 | 1.41121 | 465.51 ± 88.07^a^ | 314.66 ± 28.20^b^ | 332.30 ± 9.73^b^ |  |
| 5 | methyl-5-hepten-2-one | C110930 | C_8_H_14_O | 126.2 | 982.9 | 542.555 | 1.1746 | 118.80 ± 18.41^c^ | 244.47 ± 24.08^a^ | 163.75 ± 4.66^b^ |  |
| 6 | oct-1-en-3-ol | C3391864 | C_8_H_16_O | 128.2 | 976.2 | 528.296 | 1.15685 | 166.56 ± 23.87^a^ | 101.76 ± 7.08^b^ | 89.59 ± 6.33^c^ |  |
| 7 | Benzaldehyde-M | C100527 | C_7_H_6_O | 106.1 | 954.4 | 484.461 | 1.14975 | 595.97 ± 66.51^a^ | 449.87 ± 25.35^b^ | 317.55 ± 32.71^c^ | Monomer |
| 8 | Benzaldehyde-D | C100527 | C_7_H_6_O | 106.1 | 954.7 | 484.989 | 1.46742 | 51.92 ± 7.72^a^ | 35.02 ± 3.08^b^ | 22.09 ± 3.98^c^ | Dimer |
| 9 | Heptanal-M | C111717 | C_7_H_14_O | 114.2 | 894.5 | 382.005 | 1.34142 | 499.64 ± 84.24^a^ | 353.72 ± 31.77^b^ | 404.98 ± 14.92^b^ | Monomer |
| 10 | n-Hexanol | C111273 | C_6_H_14_O | 102.2 | 864.5 | 343.066 | 1.32749 | 375.81 ± 58.40^a^ | 219.77 ± 20.75^b^ | 451.32 ± 71.58^a^ |  |
| 11 | Hexanal-M | C66251 | C_6_H_12_O | 100.2 | 782.8 | 256.356 | 1.2664 | 1326.97 ± 15.59 | 1333.60 ± 24.79 | 1282.22 ± 49.02 | Monomer |
| 12 | Hexanal-D | C66251 | C_6_H_12_O | 100.2 | 782.8 | 256.356 | 1.56043 | 3200.00 ± 322.42^a^ | 1955.17 ± 249.81^b^ | 1430.60 ± 109.93^c^ | Dimer |
| 13 | pentan-1-ol | C71410 | C_5_H_12_O | 88.1 | 755.6 | 230.216 | 1.25621 | 147.35 ± 23.77^a^ | 84.77 ± 6.60^c^ | 121.73 ± 7.45^b^ |  |
| 14 | 3-hydroxybutan-2-one-M | C513860 | C_4_H_8_O_2_ | 88.1 | 704.1 | 187.699 | 1.06925 | 660.31 ± 98.20^c^ | 899.60 ± 108.44^b^ | 1275.08 ± 79.92^a^ | Monomer |
| 15 | 3-hydroxybutan-2-one-D | C513860 | C_4_H_8_O_2_ | 88.1 | 707.8 | 190.439 | 1.33146 | 533.67 ± 19.33^c^ | 928.28 ± 18.47^b^ | 1330.65 ± 187.31^a^ | Dimer |
| 16 | 3-methylbutan-1-ol | C123513 | C_5_H_12_O | 88.1 | 725.1 | 203.993 | 1.25005 | 15.33 ± 1.10^c^ | 26.91 ± 3.23^b^ | 57.52 ± 7.91^a^ |  |
| 17 | 3-pentanol-M | C584021 | C_5_H_12_O | 88.1 | 687.9 | 176.188 | 1.1968 | 248.91 ± 42.93^a^ | 165.55 ± 26.66^b^ | 160.49 ± 8.66^b^ | Monomer |
| 18 | 3-pentanol-D | C584021 | C_5_H_12_O | 88.1 | 687.8 | 176.148 | 1.41989 | 170.48 ± 46.02^a^ | 66.32 ± 4.88^b^ | 79.14 ± 7.49^b^ | Dimer |
| 19 | 2-Pentanone-D | C107879 | C_5_H_10_O | 86.1 | 671.3 | 168.893 | 1.36722 | 249.62 ± 15.86^b^ | 292.45 ± 10.46^a^ | 147.53 ± 7.72^c^ | Dimer |
| 20 | pent-1-en-3-ol | C616251 | C_5_H_10_O | 86.1 | 664.3 | 165.918 | 0.94004 | 214.20 ± 20.13^a^ | 125.57 ± 13.57^b^ | 140.36 ± 5.69^b^ |  |
| 21 | 3-methylbutanal-M | C590863 | C_5_H_10_O | 86.1 | 645.4 | 158.08 | 1.1855 | 248.62 ± 32.08^c^ | 349.62 ± 18.91^b^ | 617.41 ± 25.64^a^ | Monomer |
| 22 | 2-methyl-1-propanol-M | C78831 | C_4_H_10_O | 74.1 | 601 | 141.149 | 1.17031 | 247.64 ± 10.97^c^ | 383.78 ± 10.42^b^ | 567.30 ± 13.44^a^ | Monomer |
| 23 | EthylAcetate | C141786 | C_4_H_8_O_2_ | 88.1 | 585.9 | 135.819 | 1.09784 | 148.03 ± 14.13^b^ | 153.21 ± 8.23^a^ | 104.21 ± 5.24^c^ |  |
| 24 | 2-butanone-M | C78933 | C_4_H_8_O | 72.1 | 562.6 | 127.981 | 1.06044 | 398.89 ± 26.01^c^ | 476.98 ± 21.40^b^ | 567.75 ± 42.50^a^ | Monomer |
| 25 | 2-butanone-D | C78933 | C_4_H_8_O | 72.1 | 559.7 | 127.041 | 1.24628 | 1421.97 ± 149.77^a^ | 1303.24 ± 77.11^b^ | 1079.31 ± 86.21^c^ | Dimer |
| 26 | acetone | C67641 | C_3_H_6_O | 58.1 | 491 | 106.588 | 1.11609 | 1572.73 ± 85.72 | 1556.78 ± 165.33 | 1377.86 ± 197.85 |  |
| 27 | 2-methyl-1-propanol-D | C78831 | C_4_H_10_O | 74.1 | 604 | 142.232 | 1.36813 | 27.66 ± 3.03^c^ | 71.57 ± 3.01^b^ | 155.37 ± 5.65^a^ | Dimer |
| 28 | 2-Pentanone-M | C107879 | C_5_H_10_O | 86.1 | 672.6 | 169.455 | 1.12139 | 135.45 ± 4.08^b^ | 167.74 ± 15.13^a^ | 121.93 ± 2.48^b^ | Monomer |
| 29 | 2-methylbutan-1-ol | C137326 | C_5_H_12_O | 88.1 | 727.9 | 206.25 | 1.2332 | 10.65 ± 1.87^b^ | 13.28 ± 1.13^b^ | 28.15 ± 3.91^a^ |  |
| 30 | 2-methylbutanal | C96173 | C_5_H_10_O | 86.1 | 651.9 | 160.735 | 1.39641 | 27.14 ± 7.18^b^ | 43.04 ± 3.29^b^ | 103.34 ± 11.25^a^ |  |
| 31 | 3-methylbutanal-D | C590863 | C_5_H_10_O | 86.1 | 638.2 | 155.206 | 1.40412 | 17.26 ± 3.76^b^ | 30.14 ± 2.33^b^ | 80.66 ± 15.36^a^ | Dimer |
| 32 | thanol | C64175 | C_2_H_6_O | 46.1 | 441.5 | 93.953 | 1.13553 | 1247.68 ± 27.39^a^ | 903.22 ± 9.81^b^ | 707.22 ± 52.80^c^ |  |
| 33 | 1,1-diethoxyethane | C105577 | C_6_H_14_O_2_ | 118.2 | 716.7 | 197.317 | 0.97617 | 106.29 ± 3.13^c^ | 165.27 ± 3.09^b^ | 189.78 ± 16.97^a^ |  |
| 34 | 3-Methyl-2-butenal | C107868 | C_5_H_8_O | 84.1 | 769.8 | 243.469 | 1.09055 | 16.38 ± 0.20^a^ | 12.08 ± 0.57^b^ | 10.96 ± 1.32^b^ |  |
| 35 | (E)-2-pentenal | C1576870 | C_5_H_8_O | 84.1 | 741.6 | 217.735 | 1.10726 | 30.28 ± 4.27^a^ | 15.96 ± 3.10^b^ | 13.56 ± 3.15^b^ |  |
| 36 | (E)-hept-2-enal | C18829555 | C_7_H_12_O | 112.2 | 951.1 | 478.129 | 1.25468 | 23.79 ± 2.63^a^ | 11.86 ± 2.28^b^ | 7.25 ± 0.71^c^ |  |
| 37 | cyclohexanone | C108941 | C_6_H_10_O | 98.1 | 888.9 | 374.119 | 1.15414 | 25.48 ± 0.97^a^ | 17.68 ± 1.12^b^ | 14.62 ± 0.83^c^ |  |
| 38 | 2-heptanone | C110430 | C_7_H_14_O | 114.2 | 882.5 | 365.731 | 1.25955 | 46.99 ± 19.59 | 31.31 ± 4.93 | 31.51 ± 1.92 |  |
| 39 | Heptanal-D | C111717 | C_7_H_14_O | 114.2 | 893.7 | 380.829 | 1.69254 | 105.83 ± 15.53^a^ | 45.86 ± 7.27^b^ | 49.51 ± 3.40^b^ | Dimer |
| 40 | 2,3-butandione | C431038 | C_4_H_6_O_2_ | 86.1 | 549.5 | 123.779 | 1.17161 | 633.00 ± 22.05^a^ | 535.04 ± 25.65^b^ | 401.62 ± 10.65^c^ |  |

RI is retention index, Rt is retention time, Dt is migration time, [RIP rel] means normalization. The same letter denotes no significant difference, and different letters indicating significant differences (*P* < 0.05).


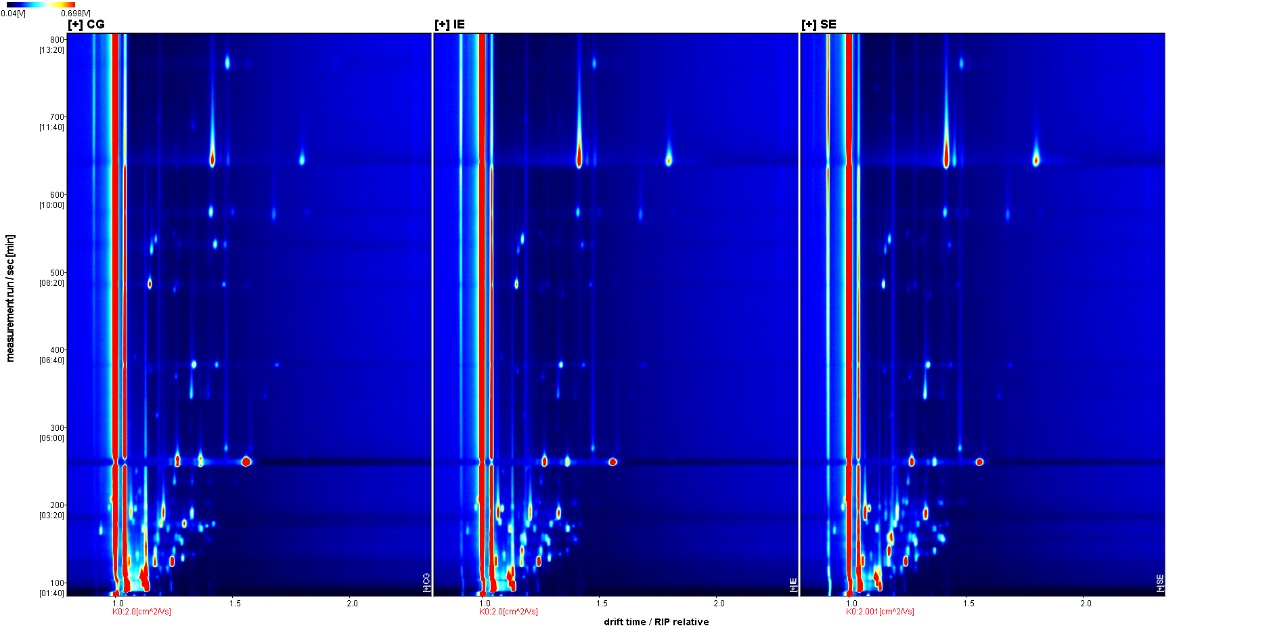


**Fig. S1** Gas-phase ion mobility spectrum of volatile compounds.


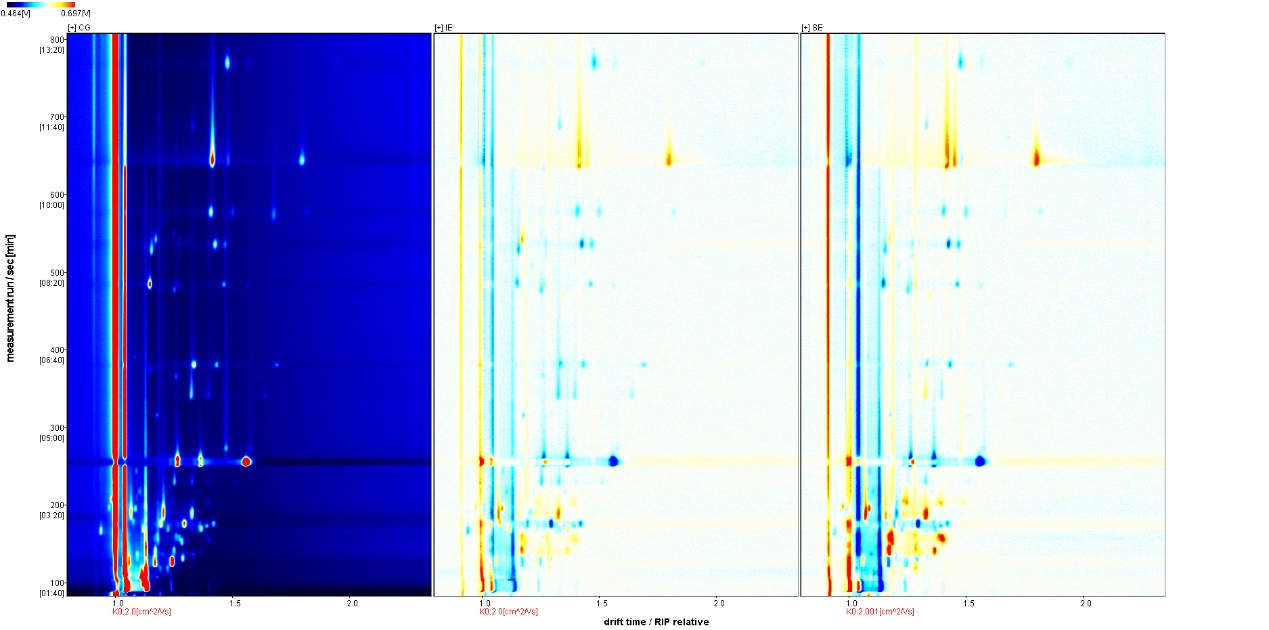


**Fig. S2** The comparison of the different GC-IMS spectrum of volatile compounds.


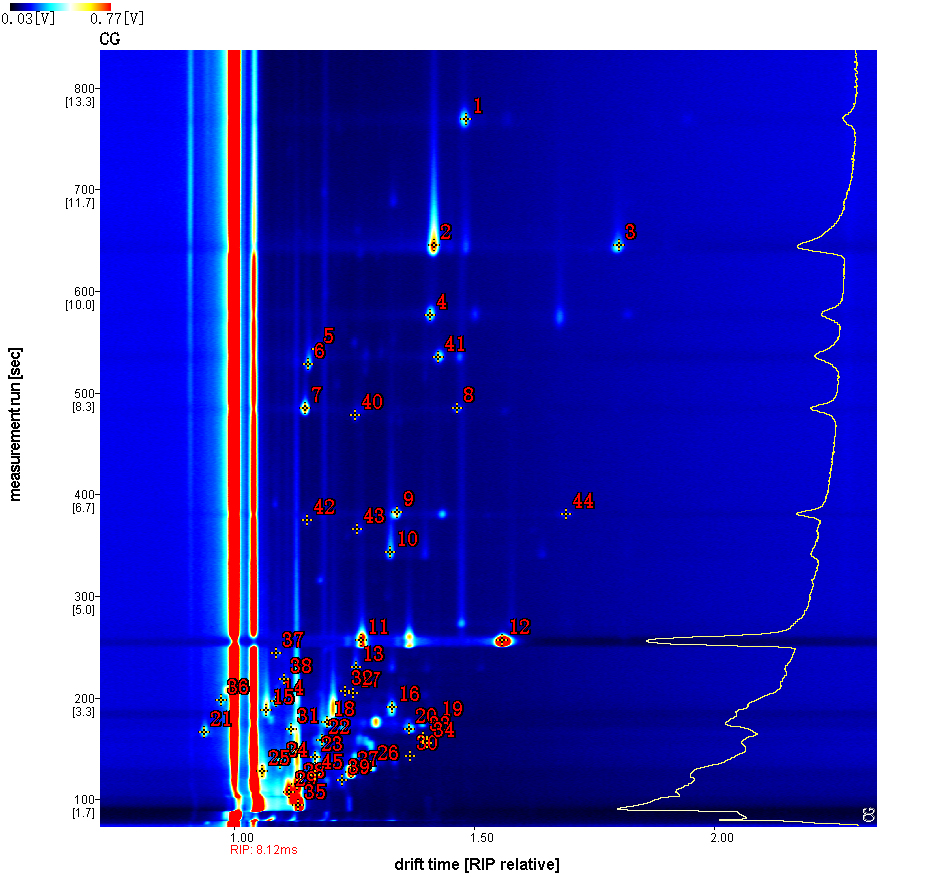


**Fig. S3** Qualitative GC-IMS spectra of volatile components in the muscles.
